# Differential Roles of Fibroblast Growth Factor Receptors (FGFR) 1, 2 and 3 in the Regulation of S115 Breast Cancer Cell Growth

**DOI:** 10.1371/journal.pone.0049970

**Published:** 2012-11-21

**Authors:** Kati M. Tarkkonen, Emeli M. Nilsson, Tiina E. Kähkönen, Julien H. Dey, Jari E. Heikkilä, Johanna M. Tuomela, Qing Liu, Nancy E. Hynes, Pirkko L. Härkönen

**Affiliations:** 1 Institute of Biomedicine, Department of Cell Biology and Anatomy, University of Turku, Turku, Finland; 2 Department of Laboratory Medicine, Tumor Biology, Lund University, Lund, Sweden; 3 Department of Biochemistry and Pharmacy, Abo Akademi University, Turku, Finland; 4 Pharmatest Services Ltd, Turku, Finland; 5 Friedrich Miescher Institute for Biomedical Research, Basel, Switzerland; 6 Turku Graduate School of Biomedical Science, Turku, Finland; 7 FinPharma Doctoral Program, Drug Discovery Section, Turku, Finland; Florida International University, United States of America

## Abstract

Fibroblast growth factors (FGFs) regulate the growth and progression of breast cancer. FGF signaling is transduced through FGF receptors 1–4, which have oncogenic or anti-oncogenic roles depending on the ligand and the cellular context. Our aim was to clarify the roles of FGFR1–3 in breast cancer cell growth *in vitro* and *in vivo*. Pools of S115 mouse breast cancer cells expressing shRNA against FGFR1, 2 and 3 were created by lentiviral gene transfer, resulting in cells with downregulated expression of FGFR1, FGFR2 or FGFR3 (shR1, shR2 and shR3 cells, respectively) and shLacZ controls. FGFR1-silenced shR1 cells formed small, poorly vascularized tumors in nude mice. Silencing of FGFR2 in shR2 cells was associated with strong upregulation of FGFR1 expression and the formation of large, highly vascularized tumors compared to the control tumors. Silencing FGFR3 did not affect cell survival or tumor growth. Overexpressing FGFR2 in control cells did not affect FGFR1 expression, suggesting that high FGFR1 expression in shR2 cells and tumors was associated with FGFR2 silencing by indirect mechanisms. The expression of FGFR1 was, however, increased by the addition of FGF-8 to starved shLacZ or MCF-7 cells and decreased by the FGFR inhibitor PD173074 in shR2 cells with an elevated FGFR1 level. In conclusion, our results demonstrate that FGFR1 is crucial for S115 breast cancer cell proliferation and tumor growth and angiogenesis, whereas FGFR2 and FGFR3 are less critical for the growth of these cells. The results also suggest that the expression of FGFR1 itself is regulated by FGF-8 and FGF signaling, which may be of importance in breast tumors expressing FGFs at a high level.

## Introduction

The fibroblast growth factor (FGF) family consists of at least 22 peptide growth factors [1] that play roles in a number of cellular processes, including proliferation, differentiation, cell survival, migration and wound healing [2]. FGFs mediate their cellular responses by binding and activating four receptor tyrosine kinases, FGF receptor (FGFR) 1–4. The extracellular ligand-binding domain of the receptors is composed of two or three immunoglobulin (Ig) -like domains that determine the ligand-binding specificity of the different FGFRs. Alternative splicing of FGFR1–3 transcripts gives rise to two isoforms, IgIIIb and IIIc, with different expression patterns and ligand-binding specificities [3]. Upon kinase activation, the phosphorylated tyrosine residues on the receptor and the FRS2 (fibroblast growth factor receptor substrate 2) adaptor protein serve as docking sites for the recruitment of SH2 (src homology-2) domains, PTB (phosphotyrosine binding) domains, adaptors, docking proteins, or signaling enzymes. A cascade of phosphorylation events further propagates the signal, eventually giving rise to cellular responses [4]. The signal transduction pathways known to be activated by FGFRs include the ERK/MAPK, PI3K and PKC/PLC pathways.

FGF-FGFR signaling results in a multitude of biological responses in different cell types. The mechanism behind how the same ligands can generate such a diversity of biological responses is not fully understood. FGF-FGFR signaling is essential in maintaining normal epithelial/stromal communication and homeostasis. Thus, the association of aberrant FGFR signaling with several human malignancies is not surprising. In breast cancer, amplification and/or overexpression of FGFR1, FGFR2 and FGFR4 have been found [5]–[7]. Moreover, FGFR2 has been recognized among the five most prominent candidate susceptibility genes in non-hereditary breast cancer [8].

In the present study, we silenced FGFR1, 2 and 3 in Shionogi 115 (S115) mouse mammary tumor cells and characterized the growth properties of the resultant cells with differential FGFR profiles *in vitro* and *in vivo*. We used fibroblast growth factor-8 (FGF-8), which is frequently overexpressed in breast and prostate cancer [9]–[13], as a ligand and FGFR activator to study the functions of FGFR1, 2 and 3.

## Results

### Silencing of FGFR Expression in the shRNA-expressing Cells

**Figure 1:**
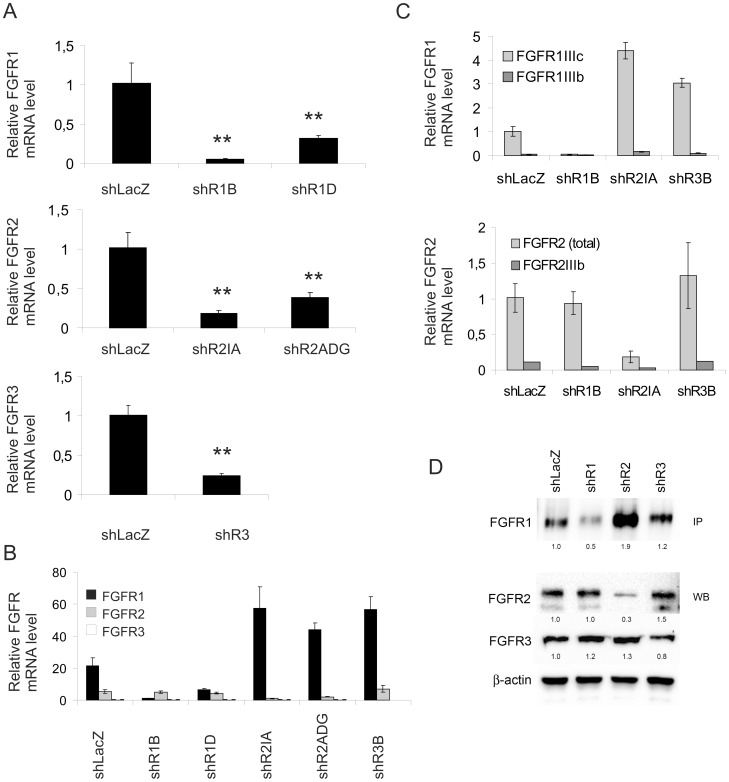
FGFR1, 2 and 3 expression in FGFR-silenced S115 cells (sh cells). Cells were grown in DMEM containing 4% iFBS and 10 nM testosterone. RNA and protein were extracted from three independently cultured sub-confluent cell plates and analyzed using qRT-PCR and immunoblotting, respectively. A) FGFR1-3 mRNA expression in the five original FGFR-silenced cell pools (shR1B, shR1D, shR2IA, shR2ADG and shR3B) compared to the expression level in control cells (shLacZ). FGFR mRNA expression is normalized to cyclophilin B expression. The statistical differences between mRNA levels were tested by non-parametric Mann-Whitney U-test, * *P*<0.05, ** *P*<0.01. B) Overall relative FGFR mRNA expression level in sh cells. C) Relative FGFR1IgIIIb/c and FGFR2IgIIIb/c mRNA expression in cells that were chosen for further studies (shR1B, shR2IA and shR3B). D) FGFR protein levels in shR1B, shR2IA and shR3B cells. Immunoprecipitation of FGFR1 was performed using 150 µg of whole cell lysates. Detection of FGFR2 and FGFR3 by western blotting was performed using 20 µg of whole cell lysates. FGFR1 expression in shR1, shR2 and shR3 cells is normalized to the protein expression in shLacZ cells. FGFR2 and FGFR3 expression in shR1, shR2 and shR3 cells are normalized to β-actin expression and thereafter to expression in shLacZ. A representative immunoblot of three independent experiments is shown.

S115 cells express FGFR1, FGFR2 and FGFR3 at high, moderate and low levels, respectively. FGFR4 is barely detectable and is not considered in this study. Each FGFR was silenced by lentiviral transfection with shRNA vectors specific for FGFR1, 2 and 3. The FGFR mRNA and protein levels were measured in the following resulting pools of puromycin-resistant cells: shLacZ control cells, shR1B, shR1D, shR2IA, shR2ADG and shR3B cells. The most efficient gene silencing was observed in shR1B cells, in which the level of FGFR1 mRNA was less than 10% of that in shLacZ cells (Figure 1A). In shR2IA and in shR3B cells, the mRNA levels of FGFR2 and FGFR3, respectively, were less than 25% of the control (Figure 1A). The relative levels of mRNA expression for FGFR1–3 in the control and each of the silenced cell pools are presented in Figure 1B. Interestingly, silencing of FGFR2 or FGFR3 led to a near 3-fold increase in the FGFR1 mRNA level (Figure 1B). To further study this finding, we silenced FGFR2 and FGFR3 using shRNA lentiviral particles in the mouse breast cancer cell line 4T1. We achieved modest downregulation (50–75% of control) of FGFR1 and FGFR3 in these cells (Figure S1A,B). Interestingly, however, in the cells with reduced FGFR2 or FGFR3, we also detected increased levels of FGFR1 mRNA (Figure S1A,B).

Based on gene silencing efficiencies, the S115 cell-derived clones shR1B, shR2IA and shR3B cells were chosen for further studies, and they were renamed as shR1, shR2 and shR3 cells. The FGFR isoforms were identified by qPCR analysis with FGFR1-3IgIIIb- and IgIIIc-specific primers. The results show that the sh-S115 cells almost exclusively express the IgIIIc isoform of FGFR1 and 2 (Figure 1C). Both FGFR3 IgIIIb and IgIIIc isoforms were detected but at very low cycle numbers (data not shown). Western blot analysis showed effective reduction of FGFR1 and FGFR2 protein in shR1 and shR2 cells, respectively (Figure 1D). Similar to the mRNA, the level of FGFR1 protein was markedly increased in shR2 cells. FGFR3 was decreased approximately to 20% in shR3 cells. Taken together, the shR1 cells represent cells with silenced FGFR1 expression and with FGFR2 and FGFR3 expression similar to control levels. The shR2 cells represent cells with reduced FGFR2 expression, associated with markedly increased FGFR1 expression compared to the control cells. The shR3 cells resemble the shLacZ cells, with only a moderate decrease in FGFR3 expression and a minor increase in FGFR1 expression.

### FGFR Silencing Affects Proliferation Rates *in vitro*

**Figure 2:**
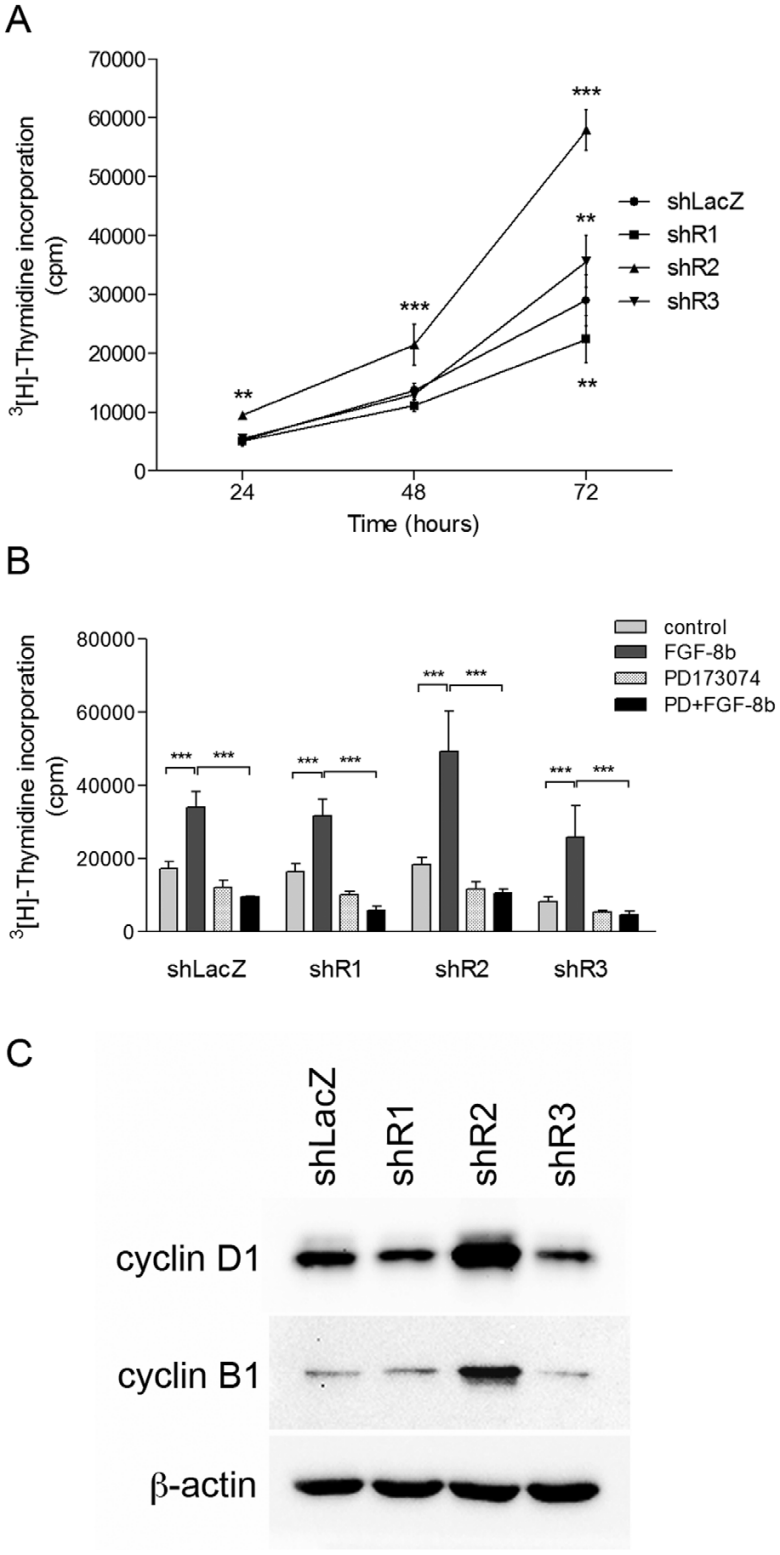
Proliferation and cyclin expression of sh cells *in vitro*. A) shLacZ, shR1, shR2 and shR3 cells were grown in full growth medium and cell proliferation was measured at 24 h intervals. Columns represent [^3^H]-thymidine incorporation, and data are expressed as mean ± SD cpm/well (*n* = 8). The experiment was repeated once, with similar results. B) Effect of the FGFR inhibitor PD173074 on FGF-8b-induced proliferation in sh cells analyzed by [^3^H]-thymidine incorporation. sh cells were pre-cultured in DC-FBS for 48 h and then treated with FGF-8b (25 ng/ml) and/or PD173074 or PBS vehicle for 48 h. The inhibitor was added 30 minutes before addition of FGF-8b. Data are expressed as mean ± SD cpm/well (*n* = 8). The statistical differences between groups were analyzed using one-way ANOVA followed by Bonferroni's multiple comparison test. * *P*<0.05, ** *P*<0.01, *** *P*<0.001. C) Cyclin D1 and cyclin B protein expression in untreated sh cells. Whole-cell lysates were generated from sh cells grown in full growth medium. Protein was subjected to SDS-PAGE and immunoblotted with antibodies against cyclin D1, cyclin B, 1 and β-actin. The experiment was repeated once, with similar results.

^3^[H]-thymidine incorporation assays showed that shR1 cells proliferated at a slower rate than shLacZ cells, whereas shR2 cells proliferated significantly faster (Figure 2A). The proliferation rate of shR3 cells was similar to that of LacZ cells. When treated with recombinant FGF-8b, all cell pools responded by increased proliferation. This response was blocked by the FGFR inhibitor PD173074 (Figure 2B). To provide mechanistic insight, we examined the level of cyclin D1 and cyclin B1 protein in the cells. Both protein levels were significantly higher in shR2 cells than in the other cells (Figure 2C), which is in accordance with a high proliferation rate of shR2 cells. The increased proliferation rate of shR2 cells was further confirmed by counting the cell number *in vitro* (Figure S2).

In contrast to S115 cells, parental 4T1 cells express relatively low levels of FGFR1 compared to FGFR2 and/or FGFR3 levels, and we could not detect alterations in the growth rates of 4T1 cells expressing FGFR2 or FGFR3 targeting shRNAs in spite of their increased FGFR1 expression (Figure S1C).

### FGFR1 Expression Level Correlates to Tumor Formation and Growth in Nude Mice

**Figure 3:**
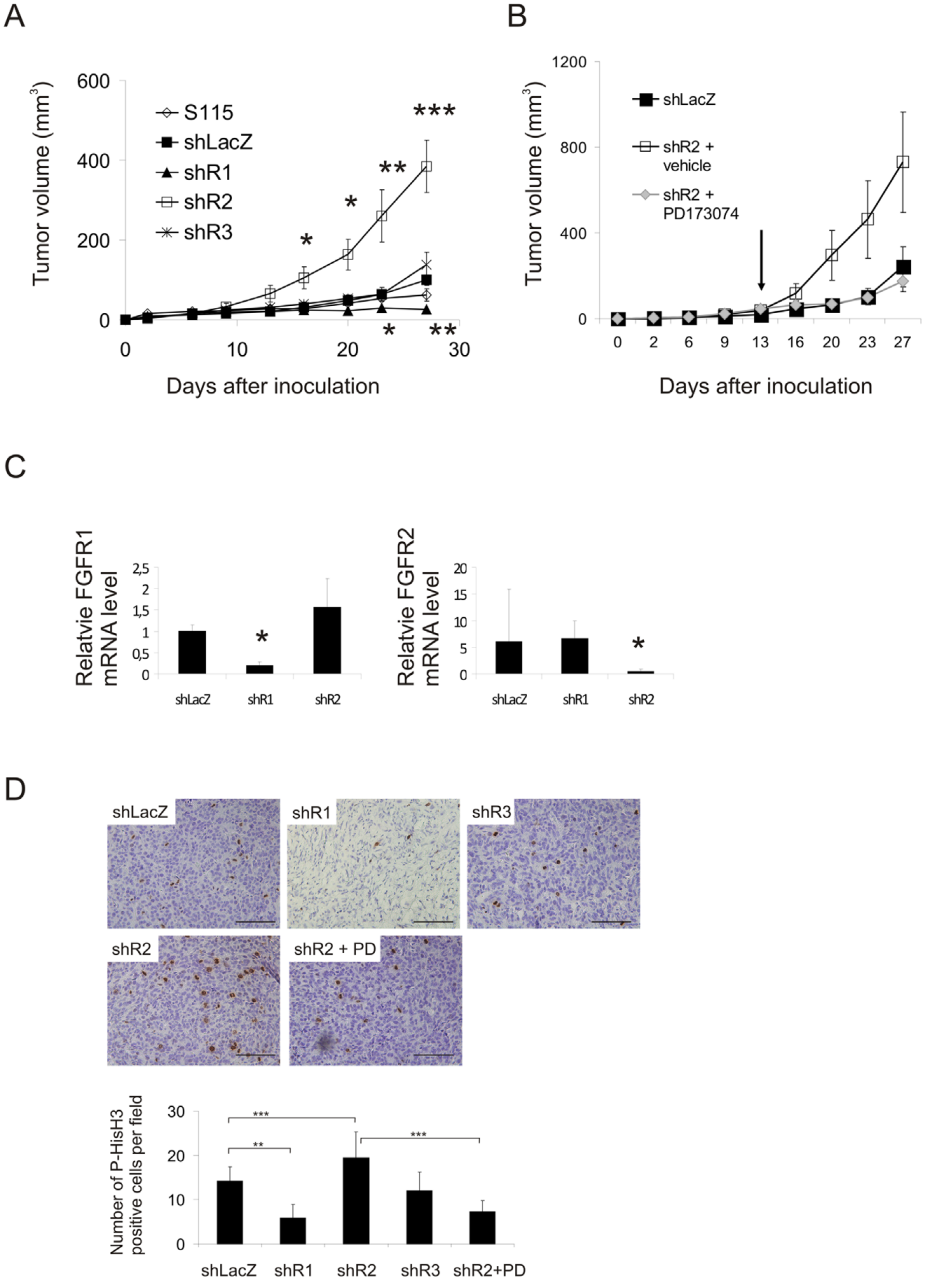
Growth of sh cells in nude mouse tumors. 1*10^6^ cells were inoculated subcutaneously (s.c.) into male nude mice (n = 5–6). A) Tumor growth was monitored every 3–4 days with a caliper until the end of the experiment. Representative growth curves from one of the two experiments with similar results are shown. Mean ± SE from each group is shown. Statistical significance of the growth rate differences were tested by Repeated Measures ANOVA, * *P*<0.05, ** *P*<0.01, *** *P*<0.001. B) FGFR inhibitor PD173074 was administered to mice bearing shR2 cell tumors, starting from day 13 (indicated by an arrow), at a dose of 25 mg/kg 5 times/week until the end of the experiment. Mean ± SE from each group is shown. C) FGFR mRNA levels were quantified by qRT-PCR analysis from shLacZ, shR1 and shR2 tumors from a separate experiment, in which the tumors were grown for 8 weeks in nude mice. Statistical difference between mRNA levels were tested by independent sample t-test, * *P*<0.05. D) P-HisH3 immunohistochemical staining of shLacZ, shR1, shR2 and shR3 tumor sections (upper panel). Scale bar = 100 µm. Lower panel shows the number of P-HisH3-positive cells per field. P-HisH3-positive cells were counted in 5–15 fields per tumor section and each group contained six tumor sections. Statistical differences between groups were tested by ANOVA followed by Bonferroni's multiple comparison test, * *P*<0.05.

The *in vivo* tumor growth of the FGFR-silenced cells was studied following their injection into nude mice. Tumor take in mice implanted with shLacZ, parental S115, shR2 and shR3 cells was 100% (n/group = 12), whereas measurable tumors formed in only 83% of the mice injected with shR1 cells (n = 12). The growth of shLacZ tumors did not differ from that of parental S115 cell tumors (Figure 3A), but there were significant differences in the growth rates of the tumors originating from different FGFR-silenced cells (Figure 3A). At 28 days, the volume of shR1 tumors was approximately one third of that of the shLacZ tumors. In contrast, the shR2 cells (with strongly increased FGFR1 levels) formed rapidly growing tumors, while the shR3 cells (with slightly increased FGFR1 and unchanged FGFR2 levels) grew only somewhat faster than the shLacZ tumors (Figure 3A). To ascertain the importance of FGFR-mediated signaling in the phenotype of the shR2 tumors, mice bearing shR2 tumors were treated with the FGFR inhibitor PD173074, starting two weeks after inoculation of the cells (Figure 3B). Tumor growth was inhibited by PD173074 but due to a variation of growth stimulation of shR2 tumors, the difference did not reach statistical significance (Figure 3B).

Because the difference between the growth rates of shR1 and shR2 cells was more marked *in vivo* than *in vitro* we wanted to confirm the expression levels of silenced FGFRs in tumors after a long-term growth *in vivo*. New subcutaneous tumors originating from shLacZ, shR1 and shR2 cells were grown for 8 weeks and FGFR1-3 mRNA levels were analysed by qPCR (Figure 3C). Although the tumors showed some variation, their profile of FGFR expression was basically similar to that *in vitro* (Figure 1) suggesting that shRNA expression leading to FGFR silencing was sustained in the tumors.

To examine proliferative activity, tumor sections were immunostained for P-HisH3 (Figure 3D). Immunostaining in shR1 tumors was very low (p<0.001), whereas the shR2 tumors showed significantly increased staining for P-HisH3 (p = 0.008) compared to the shLacZ tumors. The shR3 tumors did not show an increase in the proportion of P-HisH3 positive cells. Moreover, treatment of shR2 tumor-bearing mice with PD173074 reduced the number of proliferating cells compared to the vehicle-treated shR2 tumors (p<0.001).

### Histological Analysis of the Tumors Shows Extensive Necrosis in shR1 Tumors and a Rich Capillary Network in shR2 Tumors

**Figure 4:**
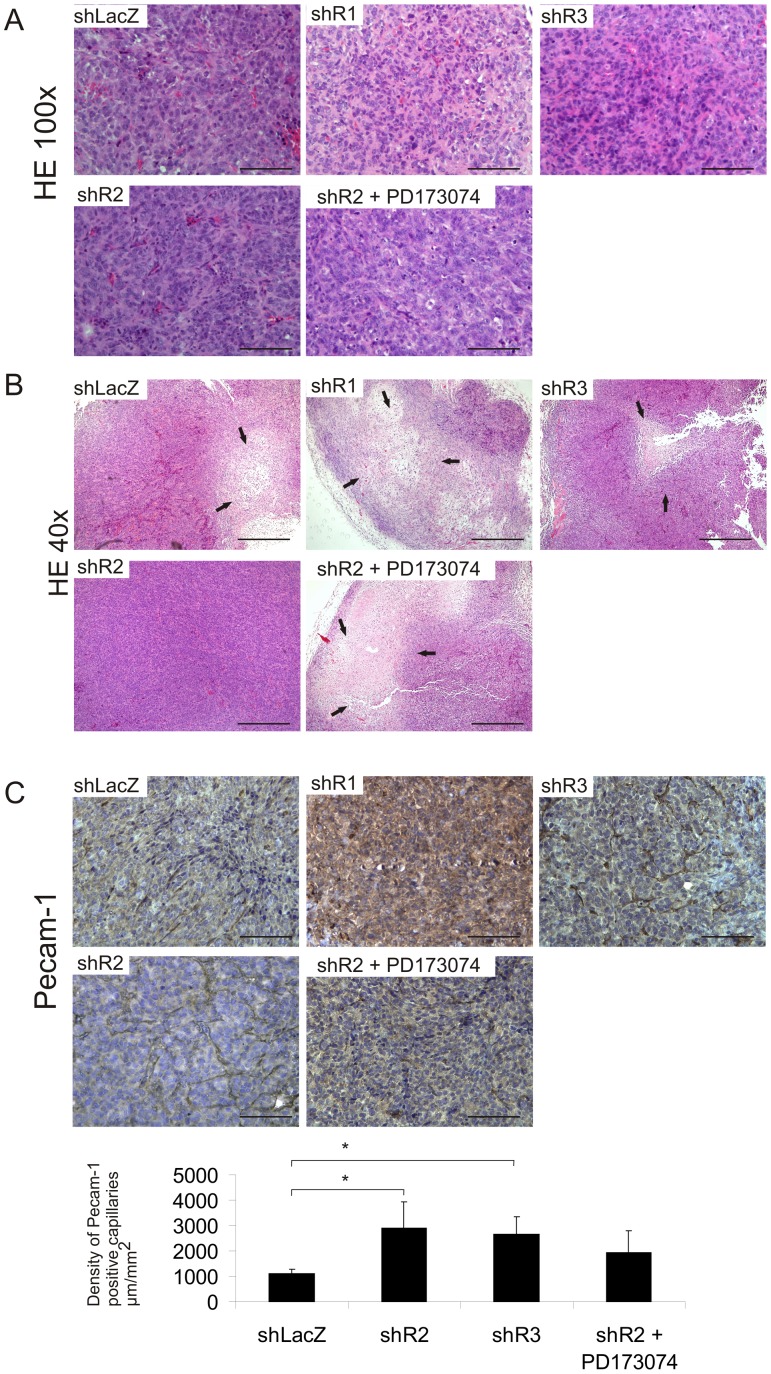
Morphology and vascularization of the sh cell tumors. A) Morphology of shLacZ, shR1, shR2 and shR3 tumors visualized by H-E staining with 100× magnification, scale bar = 100 µm and B) 40× magnification, scale bar = 500 µm. Necrotic areas are indicated by arrows. C) Vascularization in tumors was demonstrated by Pecam-1 immunoreactivity. The upper panel shows representative photomicrographs of immunohistochemical Pecam-1 staining in shLacZ, shR1, shR2 and shR3 tumors, scale bar = 100 µm. The density of Pecam-1-positive capillaries was counted in a blinded manner from 3 fields per tumor, 4–6 tumors per group and is presented as graphs (lower panel). The difference in the number of positive capillaries between the shLacZ tumors and the other tumors was tested by ANOVA followed by Bonferroni's multiple comparison test, * *P*<0.05.

The morphology of shLacZ, shR1, shR2 and shR3 tumors was visualized by H-E staining (Figure 4A, B). The shR1 tumors contained only small areas of tumor cells surrounded by necrotic and fibrotic tissue, whereas the shR2 tumors were rich in capillaries and showed little necrosis. The shLacZ and shR3 tumors were rich in capillaries, but they also contained necrotic areas. Quantification of Pecam-1 immunostained capillaries (Figure 4C) showed that the capillary density was higher in shR2 and shR3 tumors than in shLacZ tumors (p<0.05), whereas specific Pecam-1 staining in shR1 tumors was so scant that the capillaries in these tumors could not be quantified (Figure 4C). Treatment of shR2 tumor-bearing mice with PD173074 seemed to reduce vessel density, but the difference between these tumors and the untreated shR2 tumors did not reach statistical significance.

**Figure 5:**
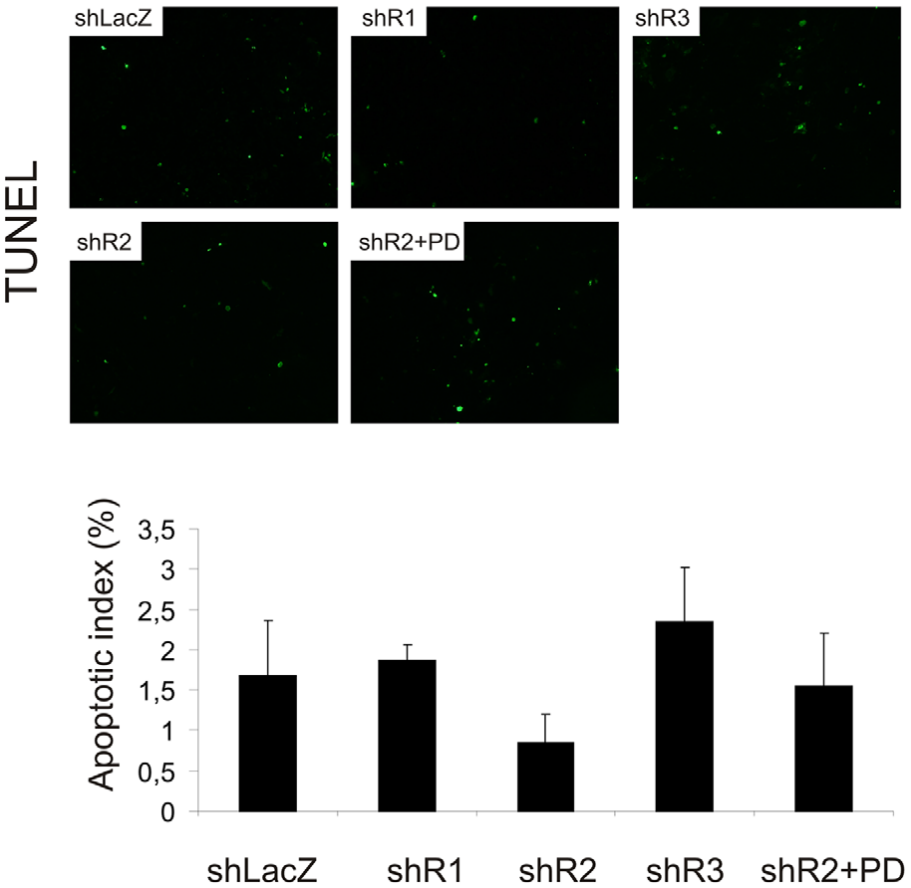
Apoptosis in sh cell tumors. Apoptosis in tumor sections was evaluated using the TUNEL assay. The number of apoptotic cells (TUNEL-stained; upper panel) and the total number of cells (DAPI-stained; not shown) were counted to determine the percentage of apoptotic cells in the tumor sections (lower panel). Cells were counted from 3 (shR1) or 6 (shLacZ, shR2, shR3) tumors per group, 5–10 random fields in each section and data are expressed as mean ± SD.

To detect apoptotic cells in the tumors, a TUNEL assay was used (Figure 5). The relative number of apoptotic cells was lower in shR2 tumors than in shLacZ tumors although the difference did not reach statistical significance after Bonferroni adjustment for multiple comparisons (p = 0.06). When the shR2 tumor-bearing mice were treated with PD173074, the number of apoptotic cells in the tumors increased to some extent. Viable areas of shR1 tumors in turn, in spite of surrounding necrotic tissue, did not show increased density of apoptotic cells, which suggests that apoptosis was not a major cause of reduced growth of shR1 tumors.

### Increased ERK1/2 Activation in shR2 Cells

**Figure 6:**
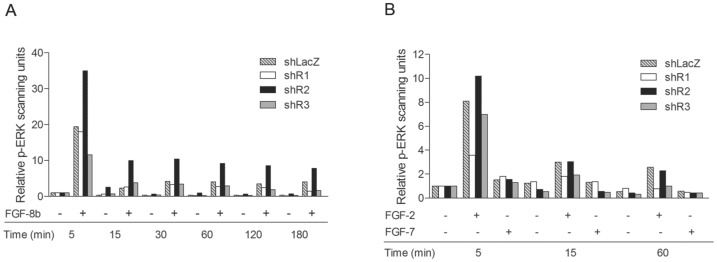
FGF-induced ERK1/2 activation in sh cells. Cells were pre-cultured in DC-FBS for 48 h and then treated with A) 25 ng/ml FGF-8b, B) 10 ng/ml FGF-2 or 100 ng/ml FGF-7 or PBS vehicle for indicated time periods. Whole-cell lysates were generated from cells and protein was subjected to SDS-PAGE and immunoblotted with antibodies against p-ERK1/2 and ERK1/2. The intensity of the bands was determined by scanning densitometry and is presented in columns as the p-ERK1/2 expression relative to ERK expression. The experiment was repeated twice with similar results.

Next, we examined activation of the ERK/MAPK and PI3K/Akt pathways in the FGFR-silenced cells. Figure 6A–B shows the quantification of P-ERK band intensities after western blotting. As shown previously for parental S115 cells [14], FGF-8b strongly stimulated ERK1/2 activation in shLacZ and shR1 cells. The signals peaked 5 min after FGF-8b addition and then rapidly decreased to low levels that were maintained over the 3-h time course period. Except for a weaker activation level, P-ERK1/2 in shR3 cells was similar to that in shLacZ and shR1 cells. In shR2 cells with high FGFR1 expression level, FGF-8b caused a 2-fold higher level of P-ERK at 5 min compared to other cells. This high level of P-ERK was sustained throughout the 3-h time course period. FGF-2 treatment caused a similar pattern of ERK phosphorylation, but the signal intensities were weaker in shR1 cells. FGF-7 binding the FGFR2 IgIIIb form caused only a very small increase in P-ERK levels, which is in accordance with a low proportion of IgIIIb forms of FGFRs in S115 cells. Immunostaining of the sh tumors showed a trend toward an increase in P-ERK in the shR2 tumors and a decrease in the PD173074 tumors in comparison to the LacZ controls; however, due to tumor tissue heterogeneity, the overall differences in tumors were difficult to evaluate (data not shown).

All cell lines showed constitutively high levels of phosphorylated Akt as also previously shown for parental S115 cells (14), and FGF-8b treatment did not cause any further increase in these levels (data not shown).

### FGFR2 Overexpression does not Affect FGFR1 Expression Directly in S115 Cells

**Figure 7:**
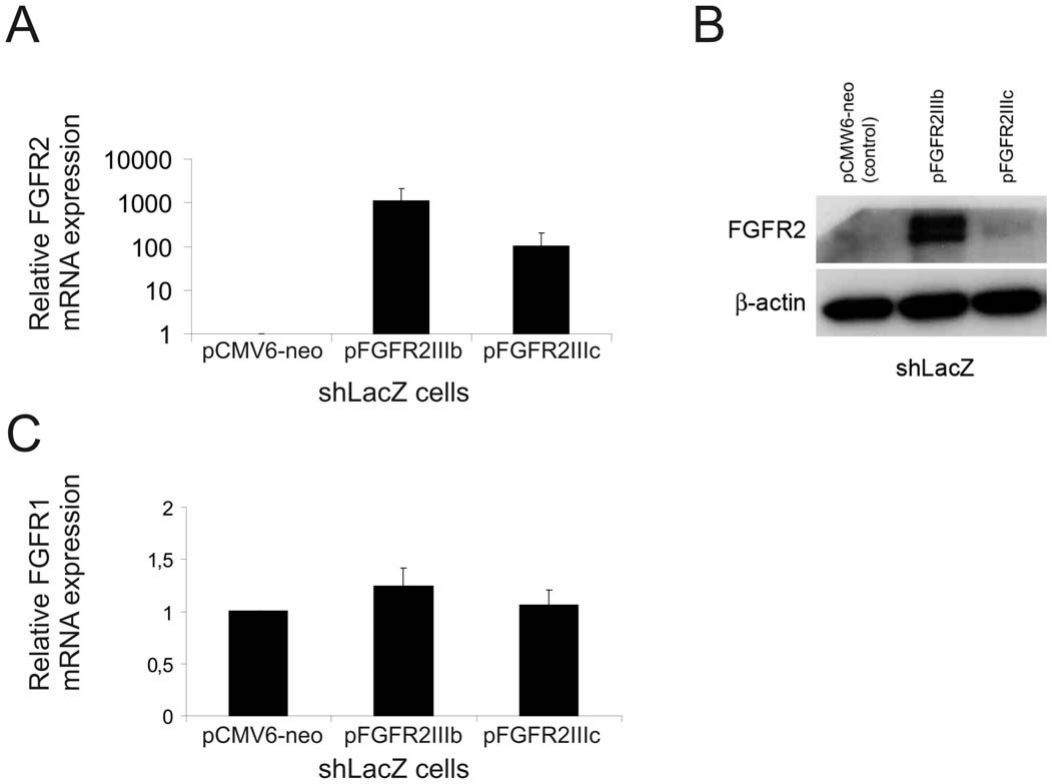
Overexpression of FGFR2IgIIIb and FGFR2IgIIIc in shLacZ cells. A) FGFR2 mRNA levels (relative to control-transfected cells (pCMV6-neo)) in FGFR2IgIIIb and FGFR2IgIIIc-transfected cells were analyzed by qRT-PCR. mRNA levels measured 24 h post-transfection are presented in a logarithmic scale. B) FGFR2 protein levels 24 h post-transfection shown by western blotting. C) Relative FGFR1 mRNA expression in FGFR2IgIIIb and FGFRIgIIIc-overexpressing cells 24 h post-transfection. The experiment was repeated twice with similar results.

Several experiments were performed to clarify the mechanism of increased FGFR1 expression in shR2 cells. Because FGFR1 upregulation was observed in FGFR2-silenced cells, we tested whether overexpression of FGFR2 would decrease FGFR1 expression. We achieved high transient overexpression of both FGFR2IgIIIb and FGFR2IgIIIc forms in shLacZ cells (Figure 7A and B), but neither form had any effect on FGFR1 mRNA (Figure 7C) or protein levels (data not shown). We also silenced FGFR2 using siRNA in S115 cells. Despite efficient downregulation of FGFR2, we did not detect changes in FGFR1 mRNA 48–72 h after FGFR2 siRNA transfection (Figure S3).

### FGF-8b Upregulates FGFR1 Expression in S115 and MCF-7 Breast Cancer Cells

**Figure 8:**
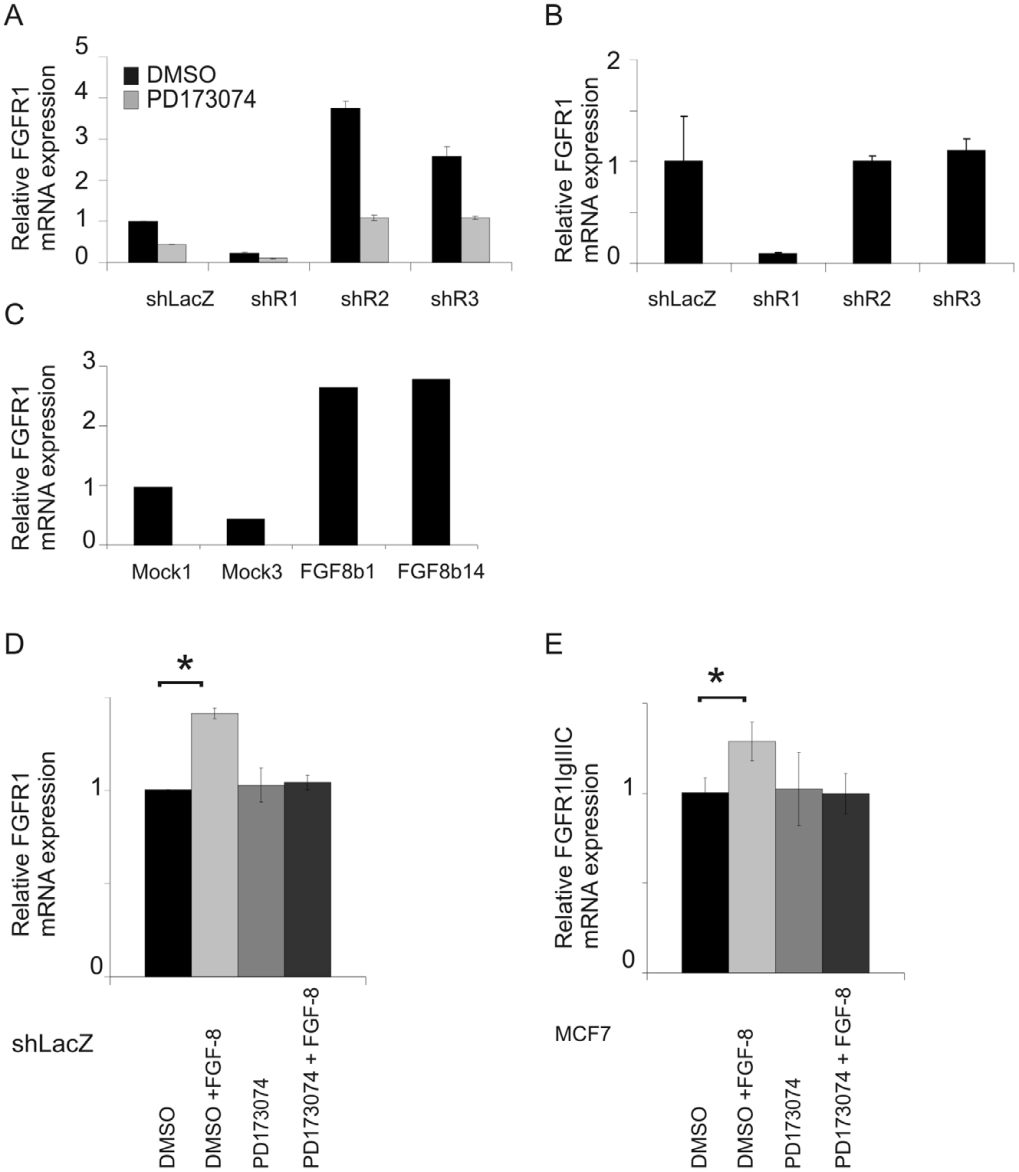
Regulation of FGFR1 mRNA expression in sh cells and MCF-7 cells. Expression of total FGFR1 or FGFR1IgIIIc was quantified by qRT-PCR in the cells cultured as follows: A) The cells grown in standard growth medium were treated with PD173074 for 24 h. B) The cells were grown without testosterone (Te) for 5 days and without serum for 48 h. C) The FGF-8b-overexpressing S115 cell lines (FGF8b1 and FGF8b14) and the transfection control cell lines (Mock1 and Mock3) were cultured in 4% DC-FBS in the absence of Te. D) shLacZ cells were grown without Te for 2 days and without serum for 24 h followed by treatment with FGF-8b (25 ng/ml) for 24 h. E) MCF-7 cells were grown in standard growth medium and treated similarly with FGF-8. The experiment was performed as triplicates and repeated twice with similar results. The statistical difference between the vehicle- and FGF-8b-treated cells was determined by independent sample t-test, * *P*<0.05.

To study whether the increased FGFR1 level in shR2 cells is related to FGF signaling, the cells were treated with PD173074 (Figure 8A). Interestingly, PD173074 down-regulated FGFR1 mRNA levels significantly in shLacZ, shR2 and shR3 cells. The effect was strongest in shR2 cells. Next, the cells were deprived of testosterone and serum to decrease autocrine and paracrine signaling by androgen-induced FGF-8 or other FGFs present in the serum. In starved cells, the FGFR1 mRNA levels decreased to the same level as in shLacZ, shR2 and shR3 cells (Figure 8B), further suggesting that FGFR1 was upregulated in shR2 and shR3 cells by FGF-8 or other FGFs included in the serum-containing growth medium. FGF-8b is the most abundantly secreted FGF in S115 cells when grown in the presence of androgens. Therefore, we analyzed FGFR1 mRNA levels in FGF-8b overexpressing S115 cell line clones [15] and found that the FGFR1 mRNA level was increased in two FGF-8b overexpressing cell lines when compared to two mock cell lines (Figure 8C). Furthermore, we tested whether exogenous FGF-8b is able to increase FGFR1 expression in serum- and testosterone-starved S115 cells. After 24 h, the FGFR1 mRNA level was significantly higher in FGF-8b-treated cells than in shLacZ control cells, and the effect could be blocked by PD173074 (Figure 8D). We also cultured human MCF-7 breast cancer cells, which do not have high endogenous FGF-8 expression, in the presence of exogenous FGF-8b. Interestingly, they showed a similar response to FGF-8b as S115 cells, suggesting that FGF-8b can upregulate FGFR1 in different types of breast cancer cells (Figure 8E).

## Discussion

Fibroblast growth factor receptors have been shown to play important roles in breast cancer [16], [17]. Activation of the FGFRs triggers similar signaling cascades in different cell types *in vitro*. However, responses of tissues to FGFR activation *in vivo* differ markedly. In order to study the role of different FGFRs in breast cancer cell proliferation and tumor growth, we individually silenced each of the endogenous FGFRs (FGFR1, 2 and 3) in S115 breast cancer cells. Effective downregulation of each FGFR was achieved in the shRNA-expressing pools of cells (shLacZ, shR1, shR2 and shR3), which showed differences in growth properties *in vitro* and *in vivo*.

Interestingly, the S115-derived shR2 cell line, which had reduced FGFR2 expression, showed highly increased expression of FGFR1. The S115-derived shR3 cells with reduced FGFR3 expression also showed some increase in the FGFR1 level compared to the shLacZ control cells. To confirm our finding we treated S115 cells with siRNA against FGFR2. FGFR2 was successfully knocked down, however, FGFR1 mRNA levels remained constant. Our results suggest that for FGFR1 regulation to occur in FGF-driven S115 cells, a stable and long-term knockdown of FGFR2 and FGFR3 is required although the mechanisms involved remain to be studied. We also treated mouse 4T1 breast cancer cells with shRNA lentiviral particles targeting FGFR1-3.The silencing efficiency was not as high as in S115 cells, but nevertheless FGFR2 and FGFR3 silencing also resulted in increased FGFR1 gene expression in these cells.

Recently, FGFR1 was reported to be a target for autoregulation by FGF-8 in neuronal cell lines [18]. Our early studies have also shown that FGFR1 levels in S115 cells are affected by FGF-2 and the heparin binding growth factor fraction (containing secreted FGF-8) of the conditioned culture medium [19]. Thus, we hypothesized that FGFR1 expression could be increased due to an altered balance of FGF signaling in FGFR-silenced cells. Our results demonstrate that the level of FGFR1 in shR2 and shR3 cells was dependent on FGF signaling because treatment with the FGFR in hibitor PD173074 or depletion of possible serum derived FGFs and endogenous FGF-8 decreased FGFR1 in shR2 and shR3 cells to the level in control shLacZ cells. We also show that when overexpressed in S115 cells or added to the culture medium of either S115 or human MCF-7 breast cancer cells, FGF-8b increased FGFR1 expression. Overexpression of FGFR2, in turn, had no effect on FGFR1 levels, suggesting that the regulation is mediated via FGFR1 itself. Taken together, our results show that FGFR1 is subject to regulation by FGF-8 (and probably also by other FGFs).

The magnitude of FGFR1 autoregulation seemed, however, to be related to the cellular level of FGFR2 (and possibly FGFR3) and the altered balance between FGFRs. The mechanisms involved remain to be explored but we speculate that post-transcriptional processing of different FGFR mRNAs is mutually regulated. This process could be affected by shRNA silencing of one FGFR. It is also possible that FGFR2 is involved in a post-transcriptional repression of FGFR1 protein synthesis. In prostate cancer cells, FGFR1 and FGF2 have recently been reported to be post-transcriptionally repressed by the microRNAs miR15 and miR16 [20]. Presently, the role of microRNAs regulated by FGFs and modulating FGF-8 and FGFR expression/signaling is poorly characterized and understood but based on the information about other receptor tyrosine kinases it is conceivable that they are also involved in the regulation of the FGFRs and their ligands in breast cancer cells. Transcriptional activation of the FGFR1 gene by E2F binding to its promoter has been shown [21]. Interestingly, a gene set enrichment analysis (GSEA) of FGF-8-controlled genes in microarray studies showed that the E2F binding motifs were highly enriched among the FGF-8 upregulated genes [22]. In fibroblasts, cyclin D1 overexpression leading to activation of the Sp1 [23] and the Rb/E2F pathways has been shown to be associated with increased FGFR1 transcription [24]. Cyclin D1 is induced by FGF signaling [14], [25], which may contribute to FGF regulation of FGFR1 expression.

The growth rate of FGFR1-silenced shR1 cells (which express primarily FGFR2) was markedly slower than that of the other cell lines *in vivo*. In contrast, the shR2 cells with silenced FGFR2 and upregulated FGFR1 grew rapidly and formed large tumors. These results suggest that while FGFR1 provides S115 cells with a strong proliferative capacity, FGFR2 is unable to promote proliferation, or may even inhibit it.

Previously, FGFR signaling has been shown to play an important role in breast cancer cell proliferation [25]. The FGFR1 gene is amplified in approximately 10% of breast cancers [26], and a correlation between amplification and FGFR1 expression levels has been examined in several studies [27]–[30]. A recent report by Turner *et al*. has shown strong evidence that FGFR1 overexpression, which is related to FGFR amplification, is a key contributor to poor prognosis in luminal-type breast cancers [31]. The role of FGFR2 in tumor development and progression appears to be more complex [32]. In genome-wide association studies [33], a particular single nucleotide polymorphism (SNP) in intron 2 of the FGFR2 gene has been associated with estrogen receptor-positive breast cancers. Recently, FGFR2 amplification and overexpression was shown to occur in 4% of triple negative breast tumors, and FGFR2 expression was found to be essential for the growth of FGFR2-amplified cell lines [34]. In contrast, FGFR2 has been shown to have a growth-limiting role, for example, in human prostate cancer cells and in hepatocellular cancer cells [35]. Interestingly, a bi-genic mouse line that has repressed FGFR2 combined with high FGFR1 activity (resembling our shR2 cells) shows enhanced tumor development in the prostate when compared to either repression of FGFR2 or overexpression of FGFR1 alone [36].

Differential signaling of FGFR1 and FGFR2 has previously been investigated in mammary epithelial cells and prostate cancer cells using drug-inducible systems, in which FGFR1 and FGFR2 can be expressed and activated at the same level [37], [38]. In these reports, ERK phosphorylation was shown to be stronger upon FGFR1 activation than upon activation of FGFR2. Moreover, FGFR1-mediated ERK activation led to increased proliferation and improved cell survival, whereas FGFR2-induced ERK activation was transient and associated with elevated apoptosis [38]. Our results also suggest that FGFR1 plays a major role in FGF-driven ERK activation because the shR2 cells expressing high FGFR1 and low FGFR2 levels responded to FGF-8b or FGF-2 by stronger and more sustained ERK1/2 activation than the cell lines expressing normal levels of FGFR2. Because ERK activation downstream of FGFRs has been associated with proliferative responses [4], this ERK1/2 activation may explain the difference in growth rates between the cell lines. Similarly, the effect of FGF-2 on ERK activation was weaker in shR1 cells, indicating that FGFR1 is also important in mediating FGF-2 responses.

As implicated above, the impact on growth after silencing FGFRs in S115 cells was much more pronounced *in vivo* than *in vitro,* suggesting the importance of the tumor environment. The fast and moderately growing shR2 and shR3 tumors, respectively, were well-vascularized, whereas the density of capillaries in the slowly growing shR1 tumors was very low. This result suggests that the angiogenic capacity of shR2 tumors is largely mediated by FGFR1, which may partly explain the strongly decreased growth rate of shR1 tumors lacking FGFR1 expression. It also suggests that the presence of FGFR2 and FGFR3 could not compensate for FGFR1 in mediation of the angiogenic effects of FGF-8 [15], [39]. Importantly, increasing evidence of non-canonical FGFR signaling that results in FGFR-mediated responses also exists [40], [41]. For example, neural CAM (NCAM) was recently shown to induce sustained FGFR1 activation [42]. Such interaction between NCAM and FGFR1 in the tumor microenvironment could potentiate the effect of FGFR1 expression and explain slow growth in the absence of FGFR1. Silencing FGFR2 and FGFR3 also increased FGFR1 mRNA levels in 4T1 cells but it did not have a significant impact on the proliferation rate of the cells suggesting that these cells are not as dependent on FGF signaling as S115 cells are.

In addition to proliferation and angiogenesis, differential FGFR expression affected tumor cell death. The shR2 tumors with high FGFR1 showed reduced apoptosis. However, although difficult to judge due to small size of tumors, FGFR1 silencing in FGFR2-expressing shR1 cells did not seem to increase cell death markedly. This suggests that although FGFR1 increases cell survival, it is not a prerequisite for survival in S115 cells. This result also suggests that FGFR2 is sufficient to protect the cells from apoptosis. Recently, FGFR-mediated PI3K activity has been shown to be crucial against apoptosis in 4T1 breast cancer cells [43]. Similarly, FGF-8b protected S115 cells from apoptosis via PI3K [14]. However, PI3K activity remained high in S115 cells under serum starvation [14], [44], and no change in P-AKT was observed in FGFR-silenced cell lines in the presence or absence of FGF-8b. Thus, either the remaining FGFRs together with autocrine/paracrine FGFs were sufficient for continuous PI3K activation or PI3K is activated by mechanisms other than those mediated by FGFRs. In either case, the sustained PI3K activity could explain the relatively low apoptotic index in all tumors.

Treating shR2 tumor-bearing mice with the FGFR inhibitor PD173074 reversed the increase in proliferation and angiogenesis to the level of control shLacZ tumors, suggesting that high FGFR1 signaling in shR2 tumors plays a crucial role in all of these responses. However, because PD173074 also inhibits VEGFR and PDGFR tyrosine kinases [16], the effect seen with PD173074 treatment may not be solely related to FGFR blockade.

In conclusion, our data show that the growth-promoting effects of FGFR1 are prominent in S115 breast cancer cells and tumors. Expression of FGFR1 itself was maintained by FGFR-mediated signaling and exposure of S115 and MCF-7 breast cancer cells to FGF-8b increased FGFR1 level. This mechanism may be of general importance in breast tumors expressing FGFs at an increased level, suggesting a dynamic regulation of FGFR level and function in the tumor environment. Considering the future promise of FGFR modulators as possible therapeutic agents, further studies on the regulation, signaling and interactions of different FGFRs in human breast cancer are highly warranted.

## Materials and Methods

### Ethics Statement

The animal experiments were carried out according to the Animal Protection Law in Finland (1076/85 and 1360/90) and the EU Directive 86/609. The experimental procedures were reviewed by the Ethics Committee on Animal Experimentation at the University of Turku and approved by the Provincial State Office of Western Finland (permission number 2008-05531).

### Preparation of shRNA Constructs

Several FGFR targeting shRNAs (5–8 for each receptor) were previously tested for the silencing effect by viral infections to 4T1 cells [45] and the most potential shRNAs were selected and used for studies in S115 cells. The pLKO.1 plasmids (Sigma, St Luis, MO) containing the desired shRNAs (1B, 1D, 2A, 2D, 2G, 2I, 3B) were purified using the PureYield Plasmid Miniprep kit (Promega, Madison, WI). A single colony per shRNA was expanded and a DNA maxi prep was performed using the Nucleobond PC-500 Kit (Macherey-Nagel, Duren, Germany) after sequencing with the following primers: 5′- caaggctgttagagagataattgga-3′ and 5′-ctttagtttgtatgtctgttgc-3′.

### Virus Production and Assay

pLKO.1 shRNA lentiviral vectors were produced by calcium-phosphate mediated co-transfection of 14.5 μg pLKO.1 siRNA (1B, 1D, 2I+A, 2A+D+G or 3B), 8.3 μg pCMVΔR8.91 and 2.1 μg pMD.G into 293T cells. Forty-eight hours later, the virus-containing media was collected and filtered (0.45 μm filter). The lentiviral titer was determined using MBA-13 cells [46]. The cells were seeded in 6-well plates. Twenty-four hours later, the diluted viral supernatant was added in the presence of 8 μg/ml polybrene and incubated for 6 h. Puromycin (3 μg/ml) was added to the medium at 48 h post-infection. After 8 days, the cells were fixed and stained with crystal violet and the colonies were counted. The titers were routinely 1–5×10^7^ cfu/ml.

### Generation of S115 Cells Stably Expressing shRNAs Against FGFR1, 2, and 3

S115 cells [47], [48] were seeded in 6-well plates. After 24 h, the lentiviral supernatant was added at an MOI (multiplicity of infection) of 100–300 together with 8 μg/ml polybrene. After incubation at 37°C for 6 h, the transduction medium was replaced with fresh medium, and the cells were incubated for 72 h before puromycin (3 μg/ml) was added. Thereafter, all puromycin-resistant cells were used as pools of shRNA-expressing cells. Prior to use, cell media were tested for the absence of replication-competent virus by measuring HIV-1 p24 antigen expression by the RETROtek HIV p24 antigen ELISA assay (ZeptoMetrix Corp., NY).

### Generation of 4T1 Cells Stably Expressing shRNAs against FGFR1, 2, and 3

The 4T1 cells were transfected by virus particles obtained from the Biomedicum center for functional genomics, University of Helsinki. The lentiviral particles of FGFR1, 2 and 3 targeting shRNA constructs created from the Sigma Mission TRC1 (The RNAi Consortium) library, 3–5 different constructs per gene, were transducted to cells similarly as to S115 cells described above.

### Cell Culture

The S115 sh-cells were maintained in DMEM supplemented with 4% heat-inactivated fetal bovine serum (iFBS) and 10 nM testosterone (standard growth medium) [49]. The 4T1 cells were maintained in RPMI-1640 medium supplemented with 10% iFBS. The estrogen dependent MCF-7 cells originate from the laboratory of Dr. C. K. Osborne (University of Texas Health Science Center, San Antonio, USA) [50] and they were maintained in RPMI supplemented with 10% iFBS, 2 mM L-glutamine, insulin (4 µg/ml) and 1 nM E_2_. For the stimulation with FGF-2, -7 and -8b (R&D Systems, Inc. Minneapolis, MN), cells were pre-cultured in DMEM supplemented with 4% DC-FBS (dextran charcoal-treated FBS). After depriving cells of testosterone for 48 h, the medium was replaced with Ham's F-12 containing bovine serum albumin (BSA; 0.2%) and mouse recombinant FGF-8b (25 ng/ml), FGF-2 (10 ng/ml) or FGF-7 (100 ng/ml) protein. The FGFR inhibitor PD173074 (Pfizer Pharmaceuticals, Ann Arbor, MI) was added 30 minutes prior to FGF-8 treatment.

### FGFR2 Transfections

The FGFR2 expression constructs SC112364 and SC111932 (here named pFGFR2IgIIIb and pFGFR2IgIIIc, respectively) were purchased from Origene (OriGene Technologies, Inc, Rockville, MD). shLacZ cells were transfected with FGFR2IgIIIb or FGFR2IgIIIc plasmids or with the vector control pCMV6-Neo using Lipofectamine 2000 (Invitrogen) according to the manufacturers’ instructions. The gene and protein expression of FGFR2 was studied 24–96 h post-transfection by qRT-PCR and western blotting, respectively.

### FGFR2 Silencing using siRNA

S115 cells were seeded in DMEM supplemented with 4% DC-FBS such that they were 30–50% confluent at the time of transfection 24 h later. Medium was replaced with Ham's F-12 containing BSA (0.2%) prior to transfection. On-Targetplus Smartpool siRNA targeting FGFR2 (Dharmacon RNA Technologies, Lafayette, CO) was diluted in OptiMEM (GIBCO) and complexed with Lipofectamin 2000 (Invitrogen) to a final concentration of 40 nM oligonucleotides. The oligomer-Lipofectamine 2000 complex was subsequently added to the medium. 48 and 72 hours after transfection cells were harvested for FGFR1 and FGFR2 mRNA expression analysis. Cells transfected with Non-Target plus siControl pool were used as a negative control.

### Cell Proliferation

Cells were seeded in 96-well plates at a density of 10^4^ cells per well in standard growth medium. The growth rate was assayed after 24, 48 and 72 h by measuring [^3^H]-thymidine incorporation as previously described [14]. For the cell number measurements, the S115 and 4T1 cells were plated at a density of 6×10^4^ cells per well in triplicates on 6-well plates. The cells were detached by trypsin and the cell number was counted by BioRad TC10 automated cell counter on the following days 1–4 from plating.

### Animals and Tumor Models

Six-week-old male nude (nu/nu) mice (Harlan, Zeist, The Netherlands) were maintained under controlled conditions. The mice were randomized into 5 groups (6 mice/group) according to body weight. Thirty minutes before the inoculation of tumor cells, an analgesic drug (Temgesic, 0.3 µg/g, Schering-Plough Nv, Brussels, Belgium) was injected subcutaneously. The mice were anesthetized by means of isoflurane inhalation (1.5–3%, air flow 200 ml/min, Univentor 400 anesthesia unit, Univentor Ltd., Zejtun, Malta). The cells were inoculated subcutaneously (1×10^6^ cells in 100 µL PBS) into the flanks of the mice, which were also implanted with a 60-d release testosterone pellet (10 mg) (Innovative Research of America, Toledo, OH). The experiment was repeated once. In the second experiment, an additional group of shR2 tumor-bearing mice was treated with PD173074. The inhibitor (25 mg/kg) was given intraperitoneally in PBS (0.1 ml) once a day, 5 days per week, beginning on day 13 and continuing until the end of the experiment. The control shR2 group was treated with vehicle (DMSO/PBS). Animal welfare was monitored daily. Tumors were measured and the tumor volume was calculated every 3–4 days [51]. The mice were sacrificed four weeks after inoculation. The tumors were excised, measured and fixed in formalin for histology and hematoxylin-eosin (H-E) staining. For FGFR mRNA analysis of the tumors, shLacZ, shR1 and shR2 cells were inoculated to nude mice as described above and the tumour tissues were collected after 8 weeks of growth, snapped-frosen in liquid nitrogen and lysed to Trizol reagent (Invitrogen, Life Technologies, Carlsbad, CA) according to manusfacturer’s instuructions.

### RNA Extraction and Quantitative Real Time RT-PCR

cDNA was synthesized from total RNA as described earlier [52]. Quantification of mRNAs was performed using the QuantiTect SYBR green real time PCR kit (Qiagen) and a DNA Engine Opticon system (MJ Research, Inc., Waltham, MA). The mRNA levels were normalized to the expression of cyclophilin B or β-actin. The results were analyzed by the 2^−ΔΔCT^–method [53]. Primer sequences used for mouse FGFR1, 2 and 3 were described by Kurosu *et al*. [54]. The primers for mouse FGFR1IgIIIb and –IgIIIc were adapted from Kettunen *et al.* [55]. The primers for FGFR2IgIIIb were 5′-ggatcaagcacgtggaaaag-3′ and 5′-actggttggcctgccctata-3′; for cyclophilin B, 5′-gggacctaaagtcacagtcaagg-3′ and 5′-gaagcgctcaccatagatgc-3′; for human β-actin, 5′-ctgtggggcgccccaggcacca-3′ and 5′-ttggccttggggttcaggggg-3′; for human FGFR1IgIIIc, 5′-gtgaatgggagcaagattgg-3′ and 5′-gcagagtgatgggagagtcc-3′.

### Immunoprecipitation and Western Blotting

For FGFR protein analyses, cells were harvested after culture in standard growth medium or after FGF treatments as described above. Western blotting was performed as described in Nilsson *et al*., 2009 [14]. For immunoprecipitation, 150 µg of whole cell lysates were incubated with FGFR1 antibodies (Abcam; ab10646; 3.8µg) and lysis buffer overnight at 4°C. The lysate-antibody complex was subsequently incubated with G-sepharose beads (Amersham Life Science) for 2 h at 4°C. The complexes were washed once with lysis buffer and twice in PBS. Proteins were separated by SDS-PAGE, transferred to polyvinylidene difluoride membranes (Millipore Corporation, Bedford, MA) and probed with primary antibodies against FGFR1 (ab53071), FGFR2, FGFR3, cyclin D1 (Abcam, Cambridge, UK), cyclin B1, phospho-ERK1/2, and Erk (Cell Signaling Technology, Beverly, MA) and β-actin (Sigma). The protein expression of P-ERK was quantified by scanning densitometry using AlphaEase FC software 4.1.0 (Alpha Innotech Corp) and is presented in graphs as expression relative to that of ERK or β-actin.

### Immunohistochemistry

Sections of formalin-fixed paraffin-embedded sh-tumors (*n* = 6) were used for immunohistochemical staining. For Pecam-1 staining, antigen retrieval was achieved by immersion of the slides in Tris/EDTA (10 mM Tris Base, 1 mM EDTA, pH 9.0). For P-HisH3 staining, antigen retrieval was performed by immersion of the slides in citrate buffer (10 mM, pH 6.0). For both antigen retrieval approaches, the slides and immersion solutions were heated in a microwave oven for 15 minutes. After washing with PBS and 0.1% Tween-20 (PBST), the sections were blocked with 1% BSA-PBST containing 10% normal serum for 30 minutes and incubated with antibodies against Pecam-1 (2.0 μg/ml; Santa Cruz, CA), P-HisH3 or P-ERK1/2 (#4370, Cell Signaling Technology) in 1% BSA-PBST at 4°C overnight.

Vascularization of the tumors was quantified by measuring the length of the Pecam-1-positive capillaries/field (3 fields/tumor from 4–6 tumors per group) using ImageJ software (ImageJ, 1.37v, Wayne Rasband, NIH, Bethesda, MD). P-HisH3 positive cells were counted manually (5–15 fields/tumor area).

### Apoptosis Assay

Apoptosis was evaluated in tumor sections by TUNEL assay using the DeadEnd Fluorometric TUNEL system (Promega). Cell nuclei were stained with DAPI by using Vectashield + DAPI (Vector Laboratories). The numbers of TUNEL-stained cells and DAPI-stained cells were counted from 3–6 tumours, 5–10 random fields in each section to determine the percentage of apoptotic cells in the tumor sections.

### Statistics

The normal distribution of the proliferation, IHC and qPCR data was tested by means of the Shapiro-Wilk W-test. The statistical differences were tested using one-way ANOVA followed by Bonferroni’s multiple comparison test (proliferation data and IHC data) or either the independent sample t-test or the non-parametric Mann-Whitney U-test (qPCR data).

The distribution of the tumor growth variables were skewed which led to log transformation for both experiments to attain normality. Repeated Measures ANOVA was performed controlling over treatment groups between time points. Individual mouse effect was taken into account with unstructured covariance structure. Degrees of Freedom were adjusted using Kenward-Roger method. Multiple comparisons between time points were adjusted using Bonferroni corrections.

## Supporting Information

Figure S1 **Silencing of FGFR2 or FGFR3 in 4T1 cells.** 4T1 cells were plated on 6-well plates and transfected by lentiviral shRNA particles against FGFR1, FGFR2 and FGFR3 (from Sigma Mission TRC1 library). The puromycin resistant cell pools were analyzed for FGFR1, FGFR2 and FGFR3 mRNA expression by qRT-PCR three weeks after transfections. Silencing of FGFR2 in shR2Y cells (A, left panel) led to increased FGFR1 mRNA levels (A, right panel) and silencing of FGFR3 in shR3Y and shR3X (B, left panel) led to increased FGFR1 mRNA levels (B, right panel) when compared with controls (shNT). The RNA levels were normalized to cyclophilin B mRNA expression, and means +/− SD of two individual RNA samples are shown. The measurements were repeated twice with similar results. Altered FGFR expression levels were not associated with alterations in the *in vitro* growth of 4T1 cells. The cell numbers of shNT, shR2Y, shR3X and shR3Y were counted on culture days 1–4 and means +/− SD/culture day (three parallel wells) from three independent cell culture experiments with similar results are shown (C).
(TIF)

Figure S2 ***In vitro*** **growth of shS115 cells.** Cells were plated at a density of 6×10^4^ cells in 6-well plates in standard growth medium and the number of attached cells were counted 12 hours after plating (Day1). The cell numbers are presented as fold-differences relative to the cell numbers of Day1. The experiment was repeated 4 times with similar results, and a representative growth curve is shown. The data points represent means +/− SD of three parallel wells per time point of each cell pool. Statistical differences between shR1, shR2, shR3 and shLacZ were tested on day 4 by the independent sample t-test followed by Bonferroni's multiple comparison test, * P<0.05; NS, nonsignificant
(TIF)

Figure S3 **siRNA mediated knockdown of FGFR2 in S115 cells.** The cells were plated in 6-well plates and transfected with siRNAs targeting FGFR2 by using lipofectamin. Cells were lysed and the mRNA levels of FGFR2 and FGFR1 in control (siControl) and FGFR2 SiRNA–treated cells (siFGFR2) were analyzed by qRT-PCR 48 and 72 h post transfection. Means +/− SD of three parallel samples are presented.
(TIF)
