# Supplementary figures and images for: Differential Roles of Fibroblast Growth Factor Receptors (FGFR) 1, 2 and 3 in the Regulation of S115 Breast Cancer Cell Growth
Source: PLoS One. 2012 Nov 21;7(11):e49970. doi: 10.1371/journal.pone.0049970 (PMC3503871; doi:10.1371/journal.pone.0049970)

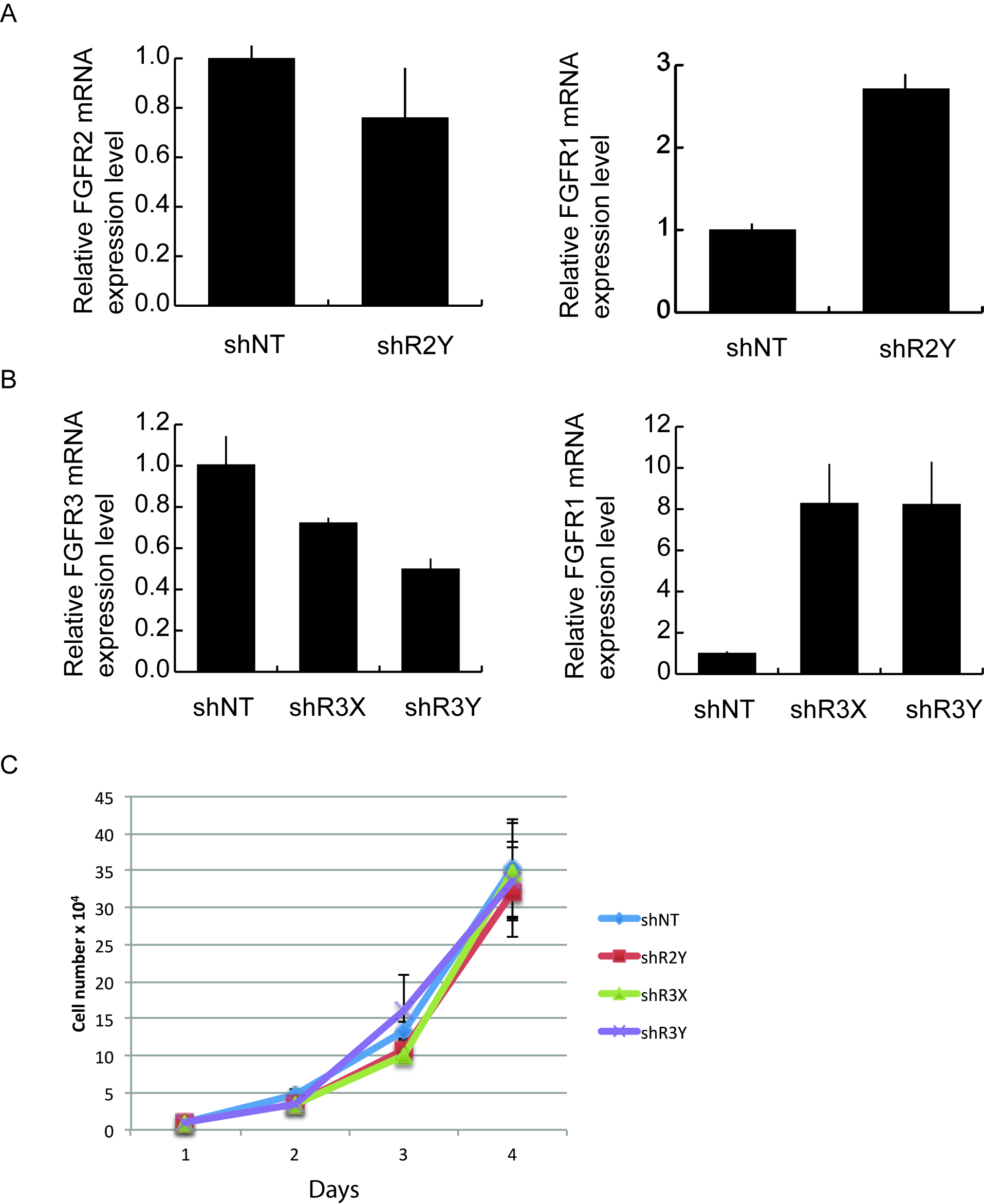

Supplement: Figure S1 — Silencing of FGFR2 or FGFR3 in 4T1 cells. 4T1 cells were plated on 6-well plates and transfected by lentiviral shRNA particles against FGFR1, FGFR2 and FGFR3 (from Sigma Mission TRC1 library). The puromycin resistant cell pools were analyzed for FGFR1, FGFR2 and FGFR3 mRNA expression by qRT-PCR three weeks after transfections. Silencing of FGFR2 in shR2Y cells (A, left panel) led to increased FGFR1 mRNA levels (A, right panel) and silencing of FGFR3 in shR3Y and shR3X (B, left panel) led to increased FGFR1 mRNA levels (B, right panel) when compared with controls (shNT). The RNA levels were normalized to cyclophilin B mRNA expression, and means +/− SD of two individual RNA samples are shown. The measurements were repeated twice with similar results. Altered FGFR expression levels were not associated with alterations in the in vitro growth of 4T1 cells. The cell numbers of shNT, shR2Y, shR3X and shR3Y were counted on culture days 1–4 and means +/− SD/culture day (three parallel wells) from three independent cell culture experiments with similar results are shown (C). (TIF) [file pone.0049970.s001.tif]

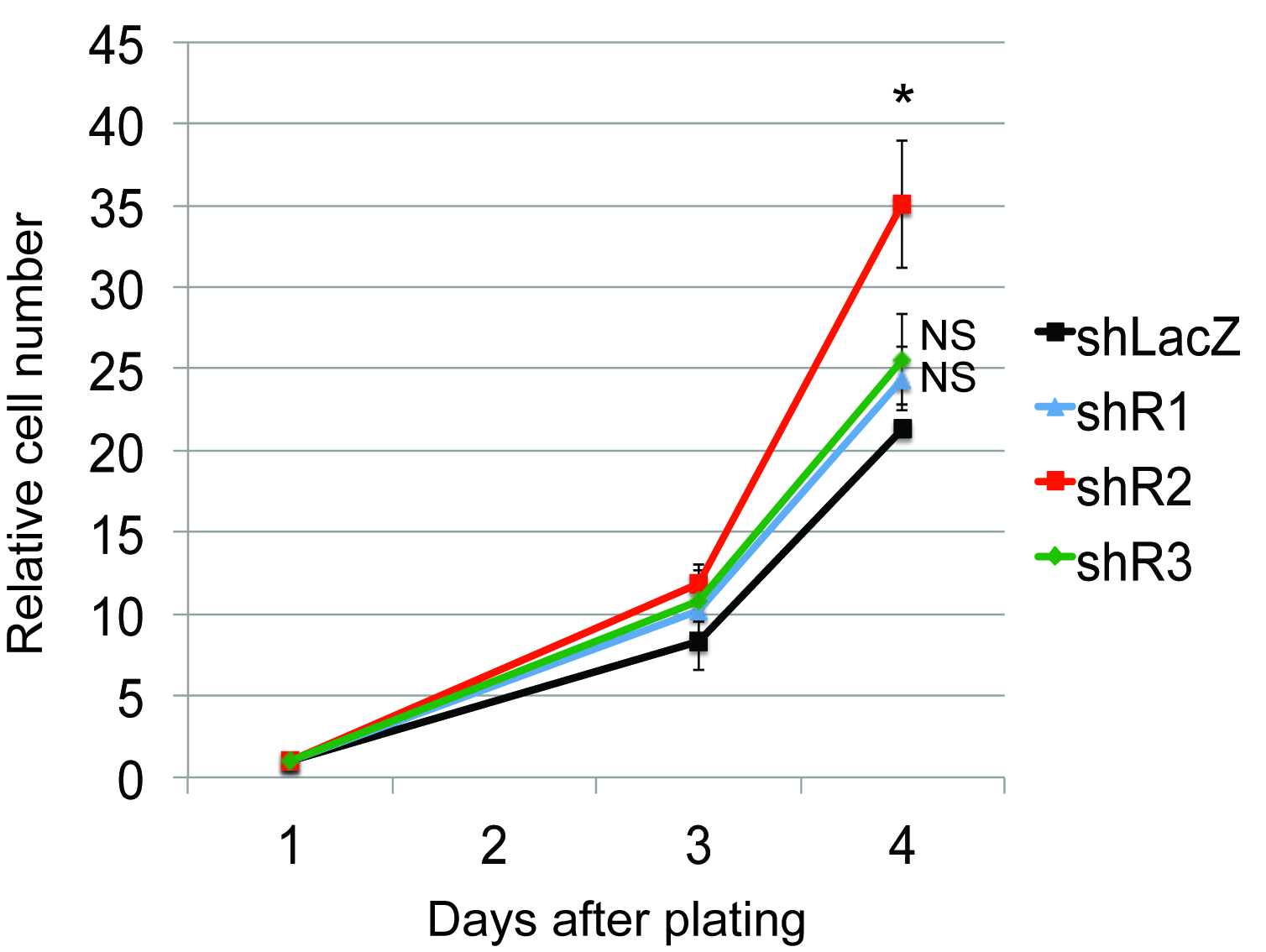

Supplement: Figure S2 — In vitro growth of shS115 cells. Cells were plated at a density of 6×10^4^ cells in 6-well plates in standard growth medium and the number of attached cells were counted 12 hours after plating (Day1). The cell numbers are presented as fold-differences relative to the cell numbers of Day1. The experiment was repeated 4 times with similar results, and a representative growth curve is shown. The data points represent means +/− SD of three parallel wells per time point of each cell pool. Statistical differences between shR1, shR2, shR3 and shLacZ were tested on day 4 by the independent sample t-test followed by Bonferroni's multiple comparison test, * P<0.05; NS, nonsignificant (TIF) [file pone.0049970.s002.tif]

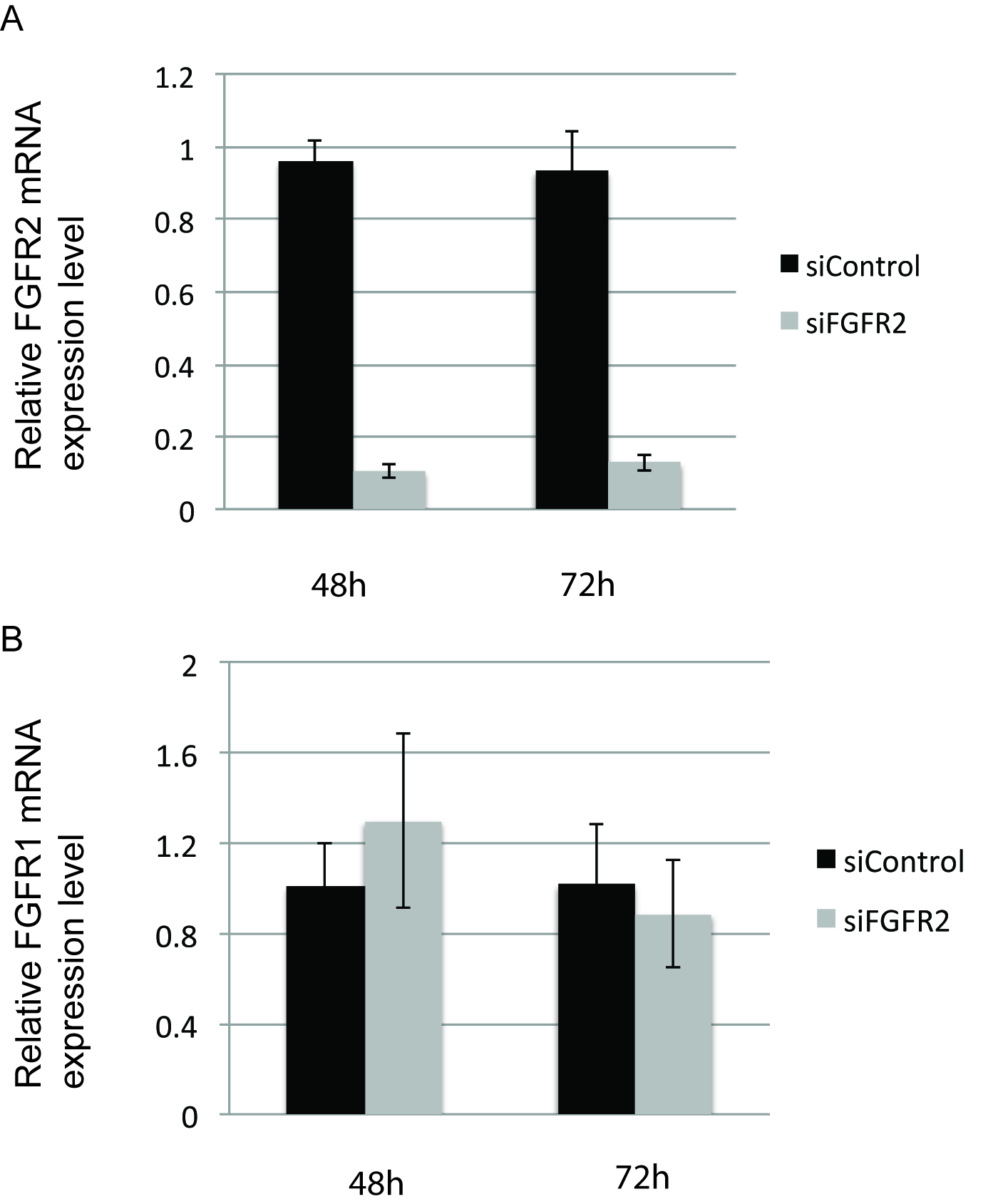

Supplement: Figure S3 — siRNA mediated knockdown of FGFR2 in S115 cells. The cells were plated in 6-well plates and transfected with siRNAs targeting FGFR2 by using lipofectamin. Cells were lysed and the mRNA levels of FGFR2 and FGFR1 in control (siControl) and FGFR2 SiRNA–treated cells (siFGFR2) were analyzed by qRT-PCR 48 and 72 h post transfection. Means +/− SD of three parallel samples are presented. (TIF) [file pone.0049970.s003.tif]
